# Immune checkpoint inhibitors–related encephalitis in melanoma and non-melanoma cancer patients: a single center experience

**DOI:** 10.1007/s00520-021-06331-5

**Published:** 2021-06-12

**Authors:** A. Taliansky, O. Furman, M. Gadot, D. Urban, J. Bar, R. Shapira-Frumer, B. Kaufman, N. Asher, R. Leibowitz-Amit, A. Itay

**Affiliations:** 1 Institute of Oncology, Sheba Medical Center at Tel HaShomer, Ramat Gan, Israel; 2 Tel Aviv University Medical School, Tel Aviv, Israel

**Keywords:** Autoimmune encephalitis, Immune checkpoint inhibitor–related encephalitis, Immune checkpoint inhibitor adverse event

## Abstract

**Background:**

Treatment with immune checkpoint inhibitors (ICI) has greatly improved survival for patients with a number of malignant diseases in recent years. Neurological immune-related adverse events (n-irAE) of varying severity have been reported in the literature. We aimed to identify the incidence of n-irAE, focusing on immune-related encephalitis (IRE), in patients treated with ICI for multiple non-hematological malignancies in our institution.

**Methods:**

All patients with histologically verified cancer that received treatment with ICI at the Sheba Medical Center between January 2017 and August 2019 were surveyed. Medical records for each patient were reviewed and information regarding n-irAE was recorded.

**Results:**

In total, 1993 patients were included. Eleven cases of IRE were recorded, affecting 0.55% of patients overall, eight had non-melanoma cancer. Eight patients had made a full recovery.

**Conclusions:**

IRE is a n-irAE more frequent than previously reported, particularly in non-melanoma patients. The diagnostic criteria and optimal treatment needs to be determined. ICI re-challenge after IRE can be considered for selected patients.

## Introduction

Immunotherapy by checkpoint inhibitors (ICI) represents a great step forward in anticancer treatment. Numerous cancer types are treated by ICI, either as monotherapy or in combinations with different kinds of agents: tyrosine kinase inhibitors (TKIs), chemotherapies, different ICI, and radiotherapy [1].

Although ICI is very successful in eliminating cancer by promoting immune response against tumor-related antigens, the toxicity of ICI treatments is second only to its efficacy. Increased T cell and other parts of immune response activation by ICI therapy leads to immune-related adverse events (irAEs) that are frequent and lead to severe adverse events in a sizable proportion of patients (10–90% in recent surveys) with fatal events occurring at 0.3–1.3% of patients [2-4].

The spectrum of systemic irAE is different for each ICI agent: the most common reported *pembrolizumab-*induced toxicities are arthropathy, hepatitis, and pneumonitis; the most common *nivolumab-*induced toxicity is endocrinopathy; while *ipilimumab* mostly induces dermatological, GI, and renal toxicities [5]. In addition, there are reports that note different landscapes of toxicities according to the paradigm of ICI use: as a single agent, as combination with other ICI, or as combination with different agent classes [6–8]. The severity of adverse events seems to increase significantly in combined protocols [7-10].

The accumulated experience with ICI treatments in oncology has brought the understanding that ICI-induced toxicity usually has multi-organ impact and mostly develops during the first months of treatment [10]. In the case of anti CTLA-4 agents, the irAEs develop earlier and in a dose-dependent manner, but in case of anti PD-1 and PDl-1 agents, the irAEs develop later and in a dose-independent manner [7, 9]. The majority of irAEs are reversible and do not always demand treatment discontinuation [1, 8].

The incidence of organ damage as a side effect of ICI treatment is different across systems: dermatological toxicity, endocrinopathies, and visceral toxicity are common [7], while neurological irAEs (n-irAEs) are rare but harbor significant clinical complications [11, 12].

Several immune-related mechanisms were suggested as explanation for the pathophysiology of n-irAE [13–15]: ICI-induced anti-tumor immune response could produce cross-reactivity with CNS antigens, similarly to the pathogenesis of paraneoplastic syndromes; therapeutic antibodies could recognize their target molecules on resident CNS cells, and there is some evidence that ICI augments pre-existing CNS autoimmune responses [3].

After reviewing publications that address ICI n-irAE from the last 5 years, we conclude that while our awareness of n-irAE increased, there are different experiences of such events. Pan and Haggiagi [16] report n-irAE ranging from 1 to 6% for monotherapy and from 12 to 14% for combination therapy, based on three large works. Kao et al. 2017 [17] report 2.9% n-irAE on anti PD-1 treatment only, Cuzzubbo et al. 2017 [18] report, based on literature search of prospective clinical trials, n-irAE incidence in single use of anti CTLA-4 which was 3.8%, anti PD-1 which was 6.1%, and 12% n-irAE in ICI combination. Spain et al. [8] report on a melanoma cohort with an overall incidence of n-irAE in 2.8% of patients, anti CTLA-4 1%, anti PD-1 3% and combination reaching 14% n-irAE. ASCO practice guideline for the management of irAEs [19] cited the same numbers as the Cuzzubbo group. Bruna et al. [20] report a 2% incidence of n-irAE in their multi-malignancy cohort.

Encephalopathy, diffuse or/and focal brain dysfunction, is a common clinical situation in cancer patients. The clinical picture varies and includes mental, speech, and behavioral disturbances; epileptic seizures; involuntary movements; motor and sensory abnormality; gaze and eye movement disturbances. As in others group of patients, the etiological variability of encephalopathy in cancer patients is wide: metabolic, inflammatory, toxic, tumorous, vascular, and even degenerative. In some patient, an encephalopathy has multifactorial etiology. Encephalitis is an inflammatory brain disorder with acute or subacute onset; encephalopathy is often part of the clinical picture of encephalitis.

Galmiche et al. [21] report specifically on an immune-related encephalitis (IRE) as n-irAE with an incidence of 2.3% in a melanoma cohort. The reported incidence of encephalitis as n-irAE is 0.1–0.2% of all n-irAE but reach 19% of high-grade n-irAE [18, 19].

The landscape of n-irAE is diverse and involves all parts of the nervous system. Few information exists about dissimilarity of n-irAE profile between melanoma and non-melanoma cancer in general [7] and only one, to our knowledge, recently published [22] review addressed the possibility of change in landscape of n-irAE when ICI use was extended to “non-melanoma” cancers.

We would like to call the attention of the neuro-oncology community to the change in n-irAEs related to the movement from an era of “predominantly melanoma ICI use”, where most n-irAEs were neuro-muscular disturbances [23], to possibly different patterns of incidence in non-melanoma patients [7].

We describe our experience with IRE as n-irAE in ICI-treated patients with non-hematological malignancies in our institute.

## Methods

Patients treated at the Institute of Oncology in Sheba Tel HaShomer Medical Center during 2017–2019, with ICI, were identified from electronic medical record. All patients with new neurological symptoms, that developed during ICI treatment and were recognized by treating oncologists, were discussed with or personally examined by neuro-oncology team’s neurologist and underwent appropriate diagnostic procedures, according to standard of care. If there were any acute or subacute brain dysfunctions, in these patients, they were qualified as encephalitis after ruling out of other etiologies. Patients treated for IRE were identified from the working database of the Neuro-Oncology Unit of the Institute, and their charts were manually reviewed.

### Treatment protocol

Five patients received standard therapy, while six patients were part of a clinical trial that included ICIs as the drug being investigated, or as baseline therapy with an additional investigational drug.

## Results

One thousand nine hundred ninety-three patients were treated with ICI in our hospital between the years 2017 and 2019; most of them were melanoma patients (Table 1). The profile of n-irAE according to disease, grouped as melanoma or non-melanoma, is shown in Table 4. Out of these patients, 11 were diagnosed with ICI-induced encephalitis (IRE): patients’ characteristics are presented in Table 2 and clinical presentation symptoms are described in Table 3. Four additional patients receiving ICI had multi-organ irAEs with numerous concomitant brain pathologies (brain metastasis and previous ischemic damage), and in these cases, the possibility of IRE was in the differential diagnosis. Of the 11 patients that developed IRE, four patients received ICI as monotherapy, two patients received ICI combinations, four patients were treated by combination with chemotherapy, and one patient received ICI in combination with TKI. Five patients additionally suffered from non-neurological ICI toxicities: one patient had fatigue, two patients had skin toxicity, one patient had adrenal damage, and one patient had hepatitis.

**Table 1:** Number of patients treated with ICI at Sheba Medical Center 2017–2019, by malignancy

Malignancy	N
Melanoma	1033
Lung	559
Head and neck	93
Genitourinary	133
Gynecological	73
Gastrointestinal	74
Breast	22
Sarcoma	6

**Table 2:** Patient characteristics of immune-related encephalitis (IRE) cases

Patient number	Sex	Age	Malignancy	Treatment CPI single agent/combination
1	M	70	SCLC	Anti CTLA4 + chemotherapy
2	M	87	Urothelial carcinoma	Anti PDL1
3	F	49	Uterine carcinoma	Anti CTLA4 + anti PD1
4	F	71	Breast cancer	Anti PD1 + chemotherapy
5	M	84	Melanoma	Anti PD1
6	M	59	Melanoma	Anti PD1
7	F	71	NSCLC	Anti PD1
8	M	68	NSCLC, adenocarcinoma	Anti PD1 + chemotherapy
9	F	67	NSCLC, adenocarcinoma	Anti PDl + chemotherapy
10	F	67	Melanoma	Anti PD1 + anti LAG3
11	F	73	RCC	TKI + anti PD1

**Table 3:** Clinical characteristics of immune-related encephalitis (IRE) cases

Patient number	Clinical picture	Onset days	Brain MRI	CSF LP	CSF Protein mg\dl	PNP AB	mRS onset	mRS outcome
1	Generalized epileptic event and speech disturbances	20	Normal	27	55	Negative	4	1
2	Confusional state	12	Normal	15	90	NT	5	6
3	Cerebellar ataxia, opsoclonus, tremor	9	Normal	1	80	Negative	5	3
4	Psychotic state	24	Normal	1	54	Negative	4	0
5	Confusional state, somnolence	21	Normal	0	26	NT	3	1
6	Confusional state, somnolence, headache	210	Normal	75	98	NT	3	0
7	Speech and behavioral disturbance, generalized and complex partial epileptic event	110	Abnormal	60	101	Negative	5	6
8	Confusional state and generalized epileptic event	150	Normal	1	94	Negative	5	3
9	Confusional state and sensory neuropathy	15	Normal	3	166	Negative	4	0
10	Ataxia, speech disturbances, partial seizure	11	Normal	29	105	Negative	3	0
11	Headache, confusional state	15	Normal	6	59	NT	3	1

^*^Onset of symptoms in days since ICI treatment start

*NT*, not tested; *CFS LP*, lymphocytic pleocytosis tested in CSF; *PNP AB*, araneoplastic antibodies tested in CSF; *mRS*, modified Rankin Scale

The onset of clinical picture in these cases varied from 5 to 210 days from ICI start, with a median onset time of 20 days. The neurological picture was variable (Table 3). The most common symptom was confusional state (six patients), four patients presented with epileptic events (3 generalized and 1 partial), and three patients developed speech disturbances; headache and ataxia were seen in two patients each, while psychotic state, tremor, and opsoclonus were each seen in a single patient. The grade of disability was significant; the score according to modified Rankin Scale (mRS) was 5/4/3 points in 4/3/4 of the patients, respectively. All patients had high-grade AEs. In all but one patient (N7), the brain imaging (CT and MRI) was normal; the abnormal brain MRI had multiple white and gray matter abnormalities.

CSF examinations revealed mild protein elevation in 90% of patients and lymphocytic pleocytosis in 54.5% of patients (Table 3). Paraneoplastic antibodies were tested in 7 patients, using recombinant line assay, and all were found to be negative.

All except for one patient (N2) were treated with methylprednisolone pulse (1 g/day 5 days) followed by at least 4 weeks of prednisolone 1 mg/kg with slow tapering down. Two patients were treated with a second-line treatment: one patient with plasma exchange (N7) and one patient with plasma exchange and cyclophosphamide (N3). The patients that received only high-dose steroidal treatment responded within the first 5 days.

One patient (N2) received only low-dose steroids because of his advanced age (87 years old), and this patient died in hospice care 2 months later.

The neurological outcome was as follows (Table 3): two patients (N2 and N7) died from encephalitis. The mRS score in other patients was 1/3/0 points in 3/2/4 of the patients, respectively. Two patients, N3 and N7, recurred during steroid tapering off period. Patient N3 improved again after high-dose steroid re-treatment, while patient N7 continued to deteriorate and died despite treatment.

Oncological outcome for patients that survived IRE was good in 63.6% of patients. Only four patients had disease progression. Patient N1 with SCLC progressed 56 days after ICI discontinuation, and was treated with palliative care at recurrence. Patient N3 with uterine carcinoma progressed 150 days after ICI discontinuation and was treated with additional line of chemotherapy but died from disease progression; two other melanoma patients (N5 and N6) progressed 60 and 730 days after ICI discontinuation. Both of them were treated with ICI re-challenge: previous ICI with low-dose steroids (patient N5) and different ICI (patient N6). Both patients had partial response followed by stable disease without any neurological complications.

Postmortem evaluation was not performed on any one of the patients who died from IRE or cancer.

## Discussion

Immune-related encephalitis (IRE) is not as common as other irAEs but we find that it is more frequent than previously reported. According to our data, the frequency of IRE is 0.55% and not 0.2% as was previously reported [21].

Similar to previous reports [24], 80% of our IRE cases (9/11) developed the clinical picture within 4 months from ICI treatment onset.

Analysis of our data and the currently available literature leads to several points of discussion.

First, nervous system ICI toxicity profile might be different between cancer types. Previous reports, mostly from melanoma patient cohorts, find more PNS than CNS n-irAE (e.g., Spain et al. [24] reporting 70% PNS and 30% CNS), and indeed in our melanoma patient cohort, 76% of n-irAE involved PNS (13/17). However, from our experience in non-melanoma cancer patients, 80% of n-irAE involve the CNS (17/21) (Table 4). In cohort described by Vogrig et al. [25], where most patients were with NSCLC, similar proportions were described.

**Table 4:** Neurological immune-related adverse events (ir-AE) by system

Cancer	Total patients	CNS irAEs (%)	IRE	PNS irAEs (%)
Melanoma	1033	4 (0.38)	3	13 (1.25)
Non-melanoma	960	17 (1.77)	8	4 (0.416)

*CNS* central nervous system, *IRE* immune-related encephalitis, *PNS* peripheral nervous system

The pathophysiological mechanism of ICI n-irAE and paraneoplastic syndrome could be partially similar. An analogy of the situation with different incidence of paraneoplastic syndromes in different kinds of cancers could be done. The reported incidence of neurological paraneoplastic syndromes in melanoma is low [13, 26]. At the same time, in lung (especially small cell lung cancer), gynecological malignancies are significantly higher [13, 26]. The same tendency was observed, according to our data, in distribution of the incidences of encephalitis as ICI n-irAE. Among melanoma cancer patients, the incidence of encephalitis as ICI n-irAE was relatively low in comparison to incidence of this n-irAE among non-melanoma cancer patients. In light of that, the relatively high incidence of encephalitis as ICI n-irAEs in the population of genitourinary cancer patients is surprising and will be better understood in future studies.

An additional explanation for different toxicity profile, between cancer types, could be due to different treatment approaches in different cancer types. As previously reported, the incidence of ICI irAEs is higher when ICI is used in combination with other agents. Combination of ICI with non-IPI agents is not used for treatment of melanoma cancer but they are used for the treatment of non-melanoma cancers. This approach might explain the increase in incidence of encephalitis, as ICI n-irAE, in some non-melanoma cancer patients, but not in genitourinary cancer patients.

Secondly, according to the data published by Vogrig et al. [22], paraneoplastic antibodies were positive in about 60% of patients with encephalitis as an ICI n-irAE, but other authors reported that in most patients with encephalitis, the paraneoplastic antibodies were negative with the exception of a few cases that were positive for anti NMDA antibodies [21, 27]. 63.6% of our patients were tested for paraneoplastic antibodies and were negative. The paraneoplastic antibody positivity in n-irAE patients is different probably due to different methods of testing. Evaluation of paraneoplastic antibody positivity as pre-treatment evaluation is currently not a common practice. Additionally, no consensus exists regarding the significance of follow-up, of these antibodies levels, in positive patients as a marker of treatment efficacy or oncological disease activity. Only sporadic studies addressed this topic [28]. It is possible that testing for paraneoplastic antibodies, prior to ICI therapy, will reveal the patients at risk to develop the ICI n-irAEs, even though only part of the patients that had paraneoplastic antibodies prior to ICI treatment continued to develop n-irAEs [28].

In recent years, providing a good quality of life for oncological patients, during treatment, has become an additional important treatment outcome. The neurological deficits, caused by nervous system damage, could significantly impair patients’ quality of life. They warrant increased awareness and attention of oncologists to neurological symptoms during treatment visits. The increased awareness of n-irAE might have influenced the increased number of reported cases of n-irAE.

The diagnosis of IRE remains a challenge and continues to be a diagnosis of exclusion. We did not find, in the literature, any specific (clinical, laboratory, or imaging) diagnostic criteria for IRE. As most patients with n-irAE have multi-system toxicity, it is possible that in cases with high-grade visceral toxicity, where metabolic disturbances are prominent, the signs of encephalopathy are not rare. This raises the possibility that patients diagnosed as non-neurological irAE might have nervous system toxicity as well, but accurate diagnosis was not possible. Therefore, it is possible that the real incidence of IRE is higher.

The treatment of IRE is based on experts’ opinion [11, 29] and for now is identical to non-ICI-related autoimmune encephalitis. From our experience, high-dose steroids are a sufficient treatment for the majority of patients and the response to this treatment could be a prognostic factor of recovery. The best second-line treatment and the right time for switching treatment lines are not known yet. The optimal duration of steroid treatment is also unknown. For our patients, after the completion of pulse methylprednisolone, prednisone treatment was prescribed for 1 month on average with slow tapering down.

The question of ICI re-challenge, following IRE, is very relevant. For most of our patients, IRE was a reversible condition, even though 18% of our patients died as a result of IRE (2 patients). When considering that ICI is the best or only oncological treatment, physicians need to make a calculated risk evaluation. We report that in our cohort, out of four progressed patients, two melanoma patients were treated with ICI re-challenge with no complication. Due to the small number of patients, we do not have the right tools to make a risk assessment conclusion.

## Conclusion

We conclude that the real incidence of encephalitis as ICI n-irAE is higher than was previously suggested. We further conclude that the evolution of symptoms may be rapid and life-threatening, requiring early diagnosis and treatment decisions. Some patients with ICI-induced toxicity suffer from multi-system damages, which may mask a clinical picture of IRE.

So far, the diagnosis of IRE is clinical and per exclusion, and further research is needed in order to establish diagnostic criteria, optimal intervention, and calculated re-challenge to ICI treatment.

Prospective multi center studies with multidisciplinary approach are needed in order to define preventive and diagnostic criteria and establish the best treatment of this dangerous ICI complication.

## Data Availability

All data and material are available in uploaded tables; additional data that was not used in the tables are available on demand.
